# Beyond the battlefield: an analysis of the war-related health impacts in the Gaza strip, Palestine

**DOI:** 10.3389/fpubh.2026.1739474

**Published:** 2026-02-18

**Authors:** Maha Rabayaa

**Affiliations:** 1 Department of Biomedical Sciences and Basic Clinical Skills, Faculty of Medicine and Allied Medical Sciences, An-Najah National University, Nablus, Palestine; 2 Department of Physiology, Faculty of Medicine, Bolu Abant İzzet Baysal University, Bolu, Türkiye

**Keywords:** cancer, conflict health, disabilities, emergency, humanitarian crisis, immunization, public health, reproductive health

## Abstract

In Palestine, particularly the Gaza Strip, the ongoing military aggression has triggered a complex health crisis that extends beyond visible injuries and hospital admissions. The destruction of healthcare infrastructure, loss of medical personnel, and shortages of essential supplies have exacerbated numerous underreported health challenges. Drawing upon reports from WHO and UN agencies, as well as peer-reviewed scientific literature, this perspective highlights the obscured morbidities of war, including disrupted immunization programs, menstrual supply insecurity, reproductive health complications, delayed cancer diagnosis and treatment, and the collapse of rehabilitation services for individuals with disabilities. Routine immunization, a cornerstone of neonatal survival and infectious disease prevention, has plummeted due to insecurity, displacement, and the collapse of health services, elevating the risk of vaccine-preventable outbreaks. Menstrual health, often overlooked in conflict settings, faces severe disruption. The lack of sanitary products, water, pain management, and disposal facilities increases the risk of reproductive tract infections, infertility, and considerable psychological distress. Reproductive health is under siege, with rising rates of congenital anomalies, miscarriages, and maternal complications, which are mostly associated with ongoing malnutrition, toxicant exposure, and chronic stress, reflecting the breakdown of prenatal care and violations of reproductive rights. Cancer control efforts, already constrained by limited resources, now face further setbacks due to delayed diagnoses, restricted treatment access, and the destruction of oncology services. Environmental pollution from military actions and long-term reliance on unhealthy food may elevate cancer incidence in the coming years. Meanwhile, escalating violence has led to a surge in irreversible disabilities, particularly among children. Yet rehabilitation services remain inaccessible, compounding physical and psychological suffering. Herein, global health and humanitarian actors are called urgently to expand their focus beyond acute trauma and address these obscured health burdens with urgency. A rights-based, equity-driven approach is essential to restore dignity and health justice for Palestinians living under siege.

## 1 Introduction

The Palestine-Israel conflict has been ongoing since 1948. Since then, the conflict has repeatedly escalated and has been associated with high rates of mortality, injuries, disabilities, and frequent displacement of Palestinians (1). Since the last escalation started on October 7, 2023, around 63,000 Palestinians have been killed, more than 160,000 have been injured, and tens of thousands are missing in the Gaza Strip, based on the latest reports published in January 2026 (2). These numbers are considered higher than the numbers reported in the previous escalations (3).

The war burden in Palestine extends beyond the reported mortalities and injuries; the terrible escalation of families, destruction of the healthcare centers, loss of healthcare workers, lack of nutrition and water supplies, and lack of health supplies are all exacerbating several health, social, and environmental problems (4–7). Several concerns frequently highlight the most at-risk groups, such as advanced cancer cases and patients with rare disorders, and several evacuation campaigns to nearby countries have been carried out. However, according to the latest report from the World Health Organization (WHO), these campaigns have evacuated only 2,446 patients from July 2024 to July 2025. Furthermore, the Israeli military has systematically destroyed the already fragile healthcare system in the Gaza Strip, leaving patients with survivable injuries susceptible to death due to their inaccessibility to healthcare. Moreover, many hospitals and health centers have been out of order (8). As of February 2025, 18 of Gaza’s 36 hospitals remain operational, with no single hospital fully functional. Moreover, these hospitals are working over the normal capacity, severely lacking resources and medical supplies (9). According to the 21 January 2026 WHO report, 34 out of 36 hospitals in the Gaza Strip (94%) have been damaged (10).

The above-mentioned detrimental impacts of the sustained hostilities and infrastructural collapse in Palestine could be analyzed through a three-dimensional framework of conflict evaluation. However, the war’s consequences extend far beyond this lens, extending into countless domains of suffering and burdens (4). This includes, but is not limited to, the lack of newborn vaccinations, the lack of menstrual supplies, the lack of diagnosis and treatment of cancer patients, and the improper handling of people with disabilities. As a result, the responsible local and global agencies, in addition to the humanitarian groups, have to work in cooperation to encompass the health-related effects of the armed conflict in Palestine and to provide proper and quick interventions (11).

This paper aims to shed light on the concealed effects of the war-related health consequences in the Gaza Strip to direct the responsible international and humanitarian agencies to exert more effort to provide support for the neglected groups of people, on whom the war adds more to their sufferings in the Gaza Strip. Given the chaotic and rapidly changing nature of an active conflict zone, a full systematic data review is impractical. Instead, a narrative synthesis of the most recent available humanitarian and clinical reports is necessary. This perspective analysis integrates evidence from peer-reviewed literature and reports issued by the United Nations (UN) agencies and the World Health Organization (WHO) to examine the overlooked health consequences of the military conflict in the Gaza Strip, with emphasis on immunization, menstrual insecurity, reproductive health, cancer management, and disability services.

This paper aims to synthesize evidence on the disruption of healthcare infrastructure and services in the Gaza Strip, to examine the obscured health consequences of prolonged military conflict, and to prioritize translational relevance and humanitarian urgency, aiming to inform global health stakeholders, policy advocates, and humanitarian organizations about the overlooked morbidities among the conflict-affected population in the Gaza Strip, Palestine. Conceptually, the analysis is guided by the social determinants of health, emphasizing how structural vulnerabilities, such as poverty, displacement, psychological stress, and food insecurity, intersect with conflict dynamics to exacerbate health outcomes and undermine population resilience.

## 2 Discussion

### 2.1 Immunization and infectious disease vulnerability

Newborn vaccination is consolidated in the health system in Palestine. The vaccination program ensures protection against serious communicable infections such as hepatitis B, polio, rubella, tetanus, and measles, and prevents the spread of these infections among the population (12). Additionally, neonatal immunization is part of the WHO Sustainable Development Goal (SDG) to reduce neonatal mortality, since many deaths are caused by vaccine-preventable diseases such as pneumonia and diarrhea (13).

Before the last escalation on 7 October 2023, the routine immunization rates in the occupied Palestinian territory were optimal; for example, the polio vaccination coverage was estimated at 99% in 2022. However, the destruction of the health system in the Gaza Strip, along with insecurity, lack of access to health centers, and displacement, has contributed to plummeting routine immunization rates, with increased risk of vaccine-preventable diseases (14, 15). With the ongoing devastating escalation in the Gaza Strip for around 2 years, newborns and children born since October 7, 2023, along with those born several months before the beginning of the war, are at risk of missing their routine vaccinations (16).

As a direct consequence of the weakened health system in Gaza, increased cases of many infectious diseases have emerged. WHO reported 54,866 cases of upper respiratory tract infections and over 33,551 cases of diarrhea, with more than half of the cases among children under 5 years old, within nearly 1 month of the conflict beginning on October 7, 2023 (17). Previous reports released in August 2024 revealed that more than 50,000 children were born within the first 10 months of the war, and they have not received any vaccines (18). According to UNICEF’s recent report on 5 November 2025, it was estimated that 1 in 5 children under 3 years of age are either completely non-vaccinated or have missed vaccinations because of the conflict (19). These numbers are considered significant outbreaks because the infectious nature of vaccine-preventable diseases, along with living in overcrowded conditions, renders these infections life-threatening, especially for immunocompromised individuals.

The appearance of the first polio case in Gaza within 25 years in a 10-month-old boy, who was paralyzed in the leg, in addition to reporting strains of poliovirus in six wastewater samples from environmental monitoring sites in the Gaza Strip have led to the launching of a vaccination campaign by the United Nations health agency to vaccinate 640,000 children in Gaza against polio (18, 20). The presence of a paralysis case might be associated with many other infected cases with no symptoms, because most people who have polio do not experience symptoms. However, the absence of a polio cure means that once paralysis occurs, it is usually permanent. Moreover, other infectious diseases have been reported among the displaced people, such as scabies, lice, chickenpox, and hepatitis A (21). In July 2024, more than 9,200 cases of chickenpox were reported among the displaced since the war began (22).

According to the latest WHO-UNICEF Estimates of National Immunization Coverage, 2023 Revision (WUENIC), which was completed on July 15, 2024, a marked decrease in vaccination coverage has been reported since the beginning of the conflict. The polio vaccination coverage decreased from 99% in 2022 to 89% in 2023, and the hepatitis vaccination coverage decreased from 100 to 91% (23). It should be noted that the reported statistics include the state of Palestine; however, a greater reduction in vaccination coverage is suspected in the Gaza Strip compared with other Palestinian areas. Moreover, the inaccessibility to many of the conflict-affected areas indicates even lower vaccination coverage and suggests that the incidence of vaccine-preventable infections is underreported.

Previous reports from other wars revealed the association of wars with the challenge of infectious diseases among refugees due to the devastating influence of conflicts on vaccination coverage. According to the United Nations Children’s Fund reports in 2016, nearly two-thirds of non-immunized children live in countries that are either partially or completely in conflict (24). For instance, around 70% of the polio cases reported globally between 2010 and 2016 were in areas of conflict. The Ukraine-Russian conflict was also associated with the spread of many infectious diseases among refugees, including coronavirus disease, measles, pertussis, tetanus, and poliomyelitis. The displacement of Ukrainians to other countries made the status more complex, and several vaccination campaigns have been conducted with a prioritizing concern on children’s vaccination for the most contagious diseases, such as mumps, measles, rubella, diphtheria, and polio (25).

The widespread prevalence of vaccine-preventable diseases within the areas of conflict is usually attributed to the destroyed health system, the shortage of healthcare workers, and the lack of information about the areas of displacement (26). The conflict in Syria was also associated with polio and measles outbreaks, and it was reported that polio vaccination coverage decreased from 91% in 2010 to about 60% in 2012 (27). In addition, the Iraq conflict was also associated with poor immunization coverage due to the deteriorated health system (28).

The administration of vaccines during periods of conflict is challenging to navigate. However, considering proper vaccination for children and newborns has to be a prioritized concern by the Ministry of Health in cooperation with the WHO to secure the lives of children against vaccine-preventable infections. In the Gaza Strip, all responsible parties should work together to increase awareness among families about vaccination. Moreover, proper evaluation of the vaccination status of the children, providing missed vaccines, and easing the entrance of healthcare workers from other countries could expedite the vaccination process.

### 2.2 Menstrual insecurity in conflict zones

Menstruation is an integrated part of normal female physiology that requires proper attention due to the concomitant symptoms and the need for special supplies. Recently, more global attention has been put on Menstrual Hygiene Management, and the topic has been introduced as a public health concern (29). Evidence suggests that improper menstrual health and hygiene can be associated with reproductive tract infections and infertility, and considerable psychosocial consequences (30).

During military conflict in the Gaza Strip, menstruation periods are additional conflict periods due to the lack of sanitary pads and appropriate disposal, the absence of facilities, water, and pain management remedies for a large number of females, especially those living in displacement settings (31). Furthermore, the lack of menstrual supplies, along with the absence of privacy and the lack of water sources and proper sanitation in the displacement settings, places a substantial proportion of Gazan females at high risk of developing both physical and psychological complications (32). These integrated difficulties and long-term psychological effects are generally more common among young females, females in the displacement tents, and especially among girls experiencing their periods for the first time during the war (33). The menstrual insecurity during humanitarian crises and/or emergencies had been previously reported in a systematic review of menstrual hygiene management (MHM) in low- and middle-income countries, which reported that the prevalence of lack of access to sanitary pads was 34%, and the prevalence of safe and proper sanitary pad disposal practices was 54%. Moreover, privacy issues have been reported due to the lack of appropriate shelters during crises (34). A study on displaced women in Myanmar and Lebanon revealed that there was insufficient access to safe and private facilities for MHM, coupled with displacement-induced shifts in menstrual practices by girls and women (35).

Consequently, the MHM must be integrated into the humanitarian and medical responses required during crises, including wars, political conflicts, and natural disasters (36). Further studies are still required to evaluate the needs of menstrual supplies for females in the Gaza Strip and to evaluate the health and mental consequences of their lack, for proper documentation of the lack of menstrual supplies and facilities as an emergent need during conflicts. Additionally, the health and humanitarian agencies, in addition to women’s supporting organizations, should take these struggles as priorities during and after the conflict, and consider Palestinian women within the global women’s protective laws. Future studies are required to systematically document the extent of unmet needs, quantify the health and mental health impacts of inadequate MHM, and evaluate recovery trajectories for affected women and girls. Such studies would provide the evidence base needed for health and humanitarian agencies, as well as women’s support organizations, to prioritize MHM in conflict-affected areas.

### 2.3 Congenital anomalies and reproductive health crisis

Prolonged military aggression in the Gaza Strip has precipitated a reproductive health crisis marked by an obvious rise in congenital anomalies, miscarriages, and maternal complications, according to a cross-sectional study on pregnant women and newborns (37). Maternal malnutrition, exposure to environmental toxicants, and chronic psychological stress converge to disrupt fetal development, which could be associated with increased birth defects and miscarriage rates. Reports from early February 2024 noted that there was a 300% increase in miscarriage rates in the Gaza Strip (38). The destruction of the healthcare system, blockade of essential medical supplies, and collapse of prenatal care infrastructure have converged to create conditions where safe childbirth is no longer a guarantee, but a gamble (7, 39). Drawing on inferential molecular insights into inflammation, malnutrition, and stress-mediated teratogenesis, urgent translational and policy interventions are needed (40). The systematic erosion of reproductive health services in the Gaza Strip constitutes not only a public health emergency but a violation of international humanitarian law. Addressing this crisis demands coordinated global action to restore maternal care, protect neonatal outcomes, and uphold reproductive rights under siege.

Emerging observational reports from field clinicians and humanitarian agencies indicate a sharp rise in congenital anomalies such as cardiac malformations, neural tube defects, and limb deformities, among newborns in the Gaza Strip. The United Nations Population Fund (UNFPA) estimated that one in three pregnancies is now considered high-risk, and one in five newborns is born preterm or underweight. These outcomes are not merely incidental; they are biologically plausible consequences of concurrent factors. The first is the long-term maternal malnutrition, particularly folate and micronutrient deficiencies, which disrupt normal embryonic development (41, 42). The World Health Organization reported that about 95% of pregnant and lactating women in the Gaza Strip suffer from severe food insecurity.

In addition to nutritional deprivation, environmental toxicants pose a silent yet potent threat to fetal development. The environmental toxicants, including heavy metals and combustion byproducts from explosives, are introduced into the ecosystem through repeated military assaults and persist in soil, water, and air. These agents are creating chronic exposure risks for pregnant women and developing fetuses (43). In Gaza, an earlier long-term surveillance study on reproductive health has revealed elevated levels of teratogenic metals such as lead, mercury, and cadmium in maternal hair samples (44). These toxicants interfere with fetal signaling pathways by disrupting gene expression, oxidative balance, and placental function, mechanisms implicated in neural tube defects, cardiac malformations, and skeletal anomalies (45, 46). Unlike acute exposures, these contaminants accumulate silently, often without immediate symptoms, yet exert profound effects during critical windows of embryogenesis. The lack of environmental remediation and continued exposure to war remnants render these risks not only biologically plausible but structurally inevitable in conflict zones.

Lastly, chronic stress and trauma, which dysregulate maternal cortisol and immune homeostasis, increase miscarriage and teratogenic risk, as reported by a previous prospective cohort study (47). While depression and anxiety are common mental disorders occurring during pregnancy or the post-pregnancy period, the ongoing conflict in the Gaza Strip exerts a compounded psychological burden on pregnant women, as pregnant women exposed to armed conflict face an increased risk of experiencing miscarriage, stillbirth, premature birth, and birth defects (48). These outcomes reflect not only individual vulnerability but also the systemic consequences of a prolonged humanitarian crisis. These factors, collectively exacerbated under sustained siege conditions, have transformed reproduction into a domain of warfare, imposing long-term biological and societal burdens on a population already enduring systemic deprivation.

The detrimental impact of military conflict on neonatal health in Gaza is neither new nor incidental. Historical evidence from a prior retrospective study at Shifa Hospital in the Gaza Strip documented a 39% rise in congenital abnormalities among newborns admitted to the neonatal intensive care unit and the gynecology department between 2008 and 2009 period marked by intensified hostilities (49). This alarming increase reflects more than clinical burden; it signals the erosion of reproductive health systems under siege, where maternal stress, disrupted antenatal care, and environmental exposures converge to shape adverse birth outcomes.

Reproductive Justice, which encompasses the complete physical, mental, spiritual, political, social, and economic wellbeing of women and girls, is systematically undermined in all its dimensions in the Gaza Strip (38). This crisis demands more than documentation; it requires translational urgency. Prioritizing the alignment of humanitarian responses with international protective frameworks has critical public health implications for safeguarding maternal and reproductive health in Palestine. Global stakeholders and policymakers have to deploy maternal health interventions in conflict zones, apply actual actions to protect reproductive rights under siege, and urgently construct the health system.

### 2.4 Oncological care under siege

Cancer is considered one of the leading causes of mortality worldwide, with more than 19 million new cases in 2020 and an ongoing increase in the mortality rate (50). With the absence of an absolute cancer treatment, the earlier diagnosis and proper intervention become critical for better prognostic outcomes (51). Late diagnosis is associated with a devastating prognosis and increased mortality rate. While the developed world is working on detecting earlier symptoms and biomarkers, the cancer patients in Palestine, even in the advanced stages of the disease, are still struggling to get access to the limited treatment options.

Even before the last escalation of the conflict in 2023, the available treatments for cancer in Palestine and, more specifically, in the Gaza Strip were inadequate due to the complex political and financial status (52). Previous reports showed that more than 30% of essential chemotherapy drugs are completely unavailable in Gaza, which impedes the treatment plans of more than half of the cancer patients (53). Furthermore, the absence of early screening facilities, the lack of specialized oncologists, and the low level of awareness about cancer mitigate the prognostic outcomes (54).

The latest escalation of the conflict in the Gaza Strip has had devastating effects on all chronic disease patients, including cancer patients. Cancer patients face three compounded, siege-driven challenges: delayed diagnosis, restricted access to treatment, and severe psychological stress. The destruction and profound restrictions on health facilities exacerbate the already fragile status of cancer care, leading to late detection and progression to advanced, often untreatable stages of cancer (55). Beyond physical suffering, cancer patients endure significant psychological distress, since they are living below the normal basic standards of life with continuous exposure to stress, violence, fear, hopelessness, and malnutrition (56). Taken together, these factors diminish patients’ physical and spiritual wellbeing, resulting in a marked reduction in quality of life (57).

The international community, together with humanitarian and health organizations, should coordinate efforts to alleviate the suffering of cancer patients in the Gaza Strip. Greater efforts must be made to evacuate cancer patients from the conflict areas and prioritize their treatment abroad. Delays in diagnosis and therapy contribute to disease progression, increasing the likelihood of advanced stages and higher mortality risk. Addressing these needs is essential to mitigate preventable deaths and improve the quality of life for cancer patients in humanitarian crises. Logistical restrictions on patients’ evacuation are another critical constraint that must be acknowledged when considering cancer care in the Gaza Strip. While evacuation and prioritization of treatment are urgent public health needs, stakeholders must also recognize the practical challenges posed by border closures, damaged infrastructure, and limited transport capacity. Addressing these barriers is essential to ensure that evacuation campaigns are feasible and can be translated into actionable humanitarian interventions. Furthermore, addressing the medical equipment and supplies required for cancer diagnosis should be an integral part of prospective plans to maintain the healthcare system infrastructure.

In addition, several previous reports indicated that environmental pollution increases the risk of health-related problems such as cancer and birth defects (58, 59). Consequently, it is hypothesized that the cancer incidence in the region may rise in the upcoming years because the population is continuously exposed to a polluted environment, products of the military actions, and wastes caused by the random destruction of the infrastructure, and is completely dependent on scarce and nutritionally inadequate food sources. To substantiate this hypothesis, longitudinal studies are required to assess the long-term effect of military action-driven environmental pollution on the incidence of cancer in the Gaza Strip as a conflict-affected zone.

### 2.5 Rehabilitation under perpetual neglect

War-related injury is a leading cause of morbidity, mortality, and disabilities in Palestine and could be considered a major public health problem. Based on previous studies on 2014 war-related injuries, 26% of individuals had sustained disability, and most of them had physical impairment (3).

The UN Children’s Fund (UNICEF) warned in December 2023 that Gaza is the most dangerous place in the world to be a child (60). The recent and previous escalations of the conflict in the Gaza Strip have increased the number of people with disabilities. Before 2023, there were 130,000 individuals with disabilities, among whom 98,000 were children with variable forms of disabilities, and several 1,000 more cases were added by the recent escalation, which started in October 2023 (61). The most recent UN report on 15 August 2025 revealed that the percentage of persons with disabilities had increased as a result of the excessive and premeditated use of force by the Israeli occupation forces reaching around 4,800 documented amputations of limbs since the beginning of the war in the Gaza Strip on 7 October 2025, among which 76% affecting the upper limbs and 24% the lower limbs (62).

With the increased number of war-related injuries and the lack of salvage surgeries, the number of cases with one or more limb amputations has increased greatly in the Gaza Strip, leaving an unprecedented number of individuals with psychological and physical implications (63). Rehabilitation, prosthetics, and securing proper facilities for individuals with disabilities remain the only means to sustain their quality of life and mitigate their sufferings. However, the rehabilitation capacity in the Gaza Strip suffers from both infrastructure collapse and the lack of rehabilitation specialists. Before the October 2023 conflict began, Gaza had a functional rehabilitation infrastructure in 11 hospitals and 5 primary health centers. Additional nongovernment organizations and aid agencies also provided several outpatient and community-based rehabilitation services. The devastating disruption of the hospitals, including rehabilitation centers, along with the unavailability of prosthetics and essential equipment like crutches and wheelchairs, made the rehabilitation capacity severely daunting. Moreover, the conflict has had a devastating impact on healthcare workers, with more than 885, including 47 physiotherapists, killed since the beginning of the war. The collapse of infrastructure is further exacerbated by a shortage in the rehabilitation workforce, undermining the sustainability of rehabilitation services in the Gaza Strip (61). A recent interview-based study on amputees from Gaza revealed that the amputees are enduring exacerbated physical complications, psychosocial suffering, and disrupted personal identity as a consequence of their limited access to medical care and rehabilitation, proper nutrition, and frequent displacement (64).

Lessons for rehabilitation strategies can be learned from previously conflict-affected areas such as Iraq and Afghanistan. Drawing on the United Nations Convention on the Rights of Persons with Disabilities (UNCRPD) goals and the World Health Organization Community-based rehabilitation (CBR) matrix, a large-scale CBR program was applied to Afghans with disabilities to improve their access to multiple services, such as physical therapy, assistive technology, education, and employment, and previous study revealed that the CBR programs can effectively improve the social and economic life of persons with disabilities and their families, even in a crisis context (65). Evaluations of earlier experience underscore the value that United Nations-related efforts could provoke wider participation of nations and confer international legitimacy to rehabilitation programs. Moreover, monitoring strategies in the rehabilitation programs are required to actually improve the rehabilitation of persons with disabilities (66).

The rehabilitation needs in the Gaza Strip are extremely demanding, necessitating immediate and sustained attention from international and humanitarian agencies. Rehabilitation services have to be systematically integrated into post-conflict reconstruction frameworks. Incorporation of rehabilitation needs in the infrastructure rebuilding plans, construction of specialized rehabilitation centers, and easing the entrance of rehabilitation and prosthetics specialists into the Gaza Strip have to be considered in the prospective reconstruction plans.

## 3 Conclusion

The protracted armed conflict in Palestine continues to exert multidimensional effects on civilian wellbeing, beginning with the deprivation of fundamental physiological needs, such as access to food, clean water, shelter, and safety, and extending to the overwhelming burden of war-related injuries that strain an already fragile healthcare system. These acute challenges are compounded by chronic and often neglected domains of suffering. The conflict is extremely devastating and is leaving several overlooked domains of suffering, including the disruption of the routine immunization programs, the need for menstrual supplies, the deterioration of reproductive health, the rise in birth defects, the lack of timely cancer diagnosis and treatment, and the absence of rehabilitation services for individuals with disabilities.

While the immediacy of trauma care and emergency response remains critical, humanitarian and policy frameworks require a broader, more inclusive view to address and apply urgent interventions in the non-ostensible domains of the conflict-associated health outcomes. Expanding global awareness of the complex humanitarian crisis in the Gaza Strip is not only a moral imperative but a prerequisite for evidence-based intervention. Rigorous quantitative and mixed-methods studies are critically needed to map unmet needs, identify service gaps, and inform targeted, sustainable responses across sectors. The application of coordinated, rights-based policy action will mitigate the long-term public health consequences of the conflict and ensure that the Palestinian population is afforded the dignity, protection, and care to which all civilians are entitled under international norms. The application of international protective laws represents a critical determinant that should be prioritized over political issues in military conflict zones.

## 4 Limitations

Finally, reporting the current suffering domains of the Palestinians in the Gaza Strip is required to ensure documentation and provoke humanitarian actions. This perspective study is limited by its reliance on secondary reporting from local and international agencies, supported by the available peer-reviewed literature from the current and previous escalations. Even though these reports are indispensable for tracking health system collapse, they are subject to potential reporting constraints in conflict settings; however, their rapid updates align closely with the evolving dynamics in the Gaza Strip and provide critical context for triangulation with peer-reviewed literature. Additionally, the current documentation of the ongoing health challenges in the Gaza Strip should be strengthened by future longitudinal, quantitative, and mixed-method field original studies based on evidence-based and validated tools to capture the evolving domains of suffering comprehensively and to inform targeted, sustainable interventions.
